# Effects of Different Nitrogen Forms and Exogenous Application of Putrescine on Heat Stress of Cauliflower: Photosynthetic Gas Exchange, Mineral Concentration and Lipid Peroxidation

**DOI:** 10.3390/plants10010152

**Published:** 2021-01-14

**Authors:** Jacinta Collado-González, María Carmen Piñero, Ginés Otálora, Josefa López-Marín, Francisco M. del Amor

**Affiliations:** Department of Crop Production and Agri-Technology, Murcia Institute of Agri-Food Research and Development (IMIDA), C/Mayor s/n, 30150 Murcia, Spain; mariac.pinero2@carm.es (M.C.P.); gines.oralora@carm.es (G.O.); josefa.lopez38@carm.es (J.L.-M.)

**Keywords:** cauliflower waste, combined stress, heat stress, nitrogen forms, plant nutrition, polyamines

## Abstract

This study examines the effect of the exogenous application of polyamine putrescine together with the application of different ratios of nitrate/ammonium (NO_3_^−^/NH_4_^+^), on the physiology of cauliflower subjected to heat stress. The 50:50 NO_3_^−^/NH_4_^+^ ratio was the best ratio against heat stress. As a result of the joint application of these compounds, a higher photosynthetic rate, a higher accumulation of both photosynthesis-related compounds and pigments, total proteins, and a change in the status of nutrients were obtained. Particularly, the decrease in content of calcium, chloride and sulphate in plants under heat stress is ameliorated by the ammonium effect. Additionally, it is important to highlight that cauliflower waste contains a higher content of mineral nutrients than floret cauliflower. These effects were more marked in young leaves. Furthermore, a synergistic effect for coping with heat stress between the polyamine and the nutritional treatment was observed. For this, both the application of putrescine and the feeding of plants with a 50:50 NO_3_^−^/NH_4_^+^ ratio before heat stress is proposed for the first time as an agricultural practice for increasing the thermotolerance of cauliflower cv Moonshine. On the other hand, due to the lower lipid peroxidation rate obtained in cauliflower leaves, these plants could be used for health purposes as ointments or other nutraceutical products, making the cultivation of this kind of cruciferous more sustainable.

## 1. Introduction

Cauliflower (*Brassica oleracea* L. var. Botrytis) is a cruciferous plant whose cultivation and consumption has increased dramatically in recent years after it was reported that this cruciferous was an important source of bioactive compounds with the ability to prevent and treat cardiovascular diseases and different types of cancer [1]. The consumption of this cruciferous plant is reflected in a world production, together with broccoli, of 25.2 million tons, with Spain being the fourth largest producer of these crucifers [2]. However, not everything is positive. In this sense, a large amount of this cruciferous plant becomes residue, and more than 50% of this residue is in the form of leaves, which has a high cost with negative effects for the environment. It is for these reasons that, in recent years, the possible reuse of this waste has been investigated [3,4].

Climate change is a significant threat not only from an ecological point of view. Thus, some studies have predicted that it is very possible that, before the end of this century, climate change will affect all levels of human activities [5]. An increase in global temperature between 1.5 and 5.8 °C by the end of the century could lead to a reduction in crop production of approximately 16%. Experts in that area predict that this reduced production seems too low as compared to the population increase that is expected to occur by the end of this century [6,7]. Therefore, it is crucial to increase our knowledge about thermotolerance, in order to be able to create plants that are resistant to high temperatures.

It is known that as a result of heat stress (depending on the intensity and duration), cauliflower can show wounds that range from a decrease in protein stability, or an increase in membrane fluidity, to the inactivation of mitochondrial enzymes [8]. Consequently, plants adjust their physiological and biochemical activities by altering their transcriptome, proteome, metabolome and lipidome to counteract these lesions and ensure cell survival [9,10].

Polyamines, including putrescine (Put), spermidine (Spd), and spermine (Spm), are a group of low molecular weight, polycationic, aliphatic, and nitrogenous compounds [11]. These biogenic amines are present in all living organisms [12]. Due to the cationic character of these aliphatic amines, they can bind to various negatively charged molecules such as RNA and DNA in cells, improving the synthesis of DNA, RNA, and proteins [13]. Additionally, it has been observed that polyamines also function as gatekeepers to regulate ion flow [13]. Thus, polyamines are involved in a wide range of biological processes in plants, including not only growth, plant development and senescence, but also providing protection to plant cells against various adverse conditions [14]. In fact, a stimulation of polyamine synthesis in plants can confer tolerance to stress [15]. Although the mechanism is not yet fully understood, it is known that the importance of polyamines lies above all in the fact that these biogenic amines are part of metabolic pathways that are interconnected with the formation of important metabolites involved in the plant’s response and signaling against stress [16,17]. Several previous studies have reported that the exogenous application of polyamines, especially putrescine, can increase the tolerance of crops to stress and promote the biosynthesis of bioactive compounds both in the floret and in the leaves of different plants [17,18,19].

It is known that nitrogen (N) is one of the most important mineral nutrients for plants, as it affects not only their growth, but also several of their physiological aspects such as photosynthesis, stomatal conductance, the maximum potential quantum efficiency of photosystem II and the chlorophyll content. Therefore, nitrogen fertilizers were used very frequently in the past, causing significant negative impacts on the environment, such as water pollution [20]. In recent years, attempts have been made to solve this problem with the efficient use of N fertilizers with a low amount of nitrates (NO_3_^−^). However, the problem that arises now is that the lack of nutrients could lead to less healthy crops with a lower yield [21]. It has been observed that nitrogen fertilization that provides ammonium (NH_4_^+^), aside from promoting plant growth, can also reduce the negative effects of heat stress [22,23]. However, the most appropriate NO_3_^−^/NH_4_^+^ ratio depends on the species.

The goal of the present study was to try to understand how heat, different NO_3_^−^/NH_4_^+^ ratios, and putrescine can interact in cauliflower. For this, the problem of N leaching in the context of a heat wave was addressed. Furthermore, we gained insights into the possible potential synergistic effects of ammonium and putrescine. Thus, we will able to promote the use of cauliflower waste for nutraceutical purposes and increase the sustainability of this crop.

The level of stress obtained was analyzed according to the degree of change in the photosynthesis rate, stomatal conductance, water use efficiency, chlorophyll content, chlorophyll fluorescence, lipid peroxidation, protein content, and mineral content in cauliflower leaves.

## 2. Results and Discussion

### 2.1. Gas Exchange

**Figure 1:**
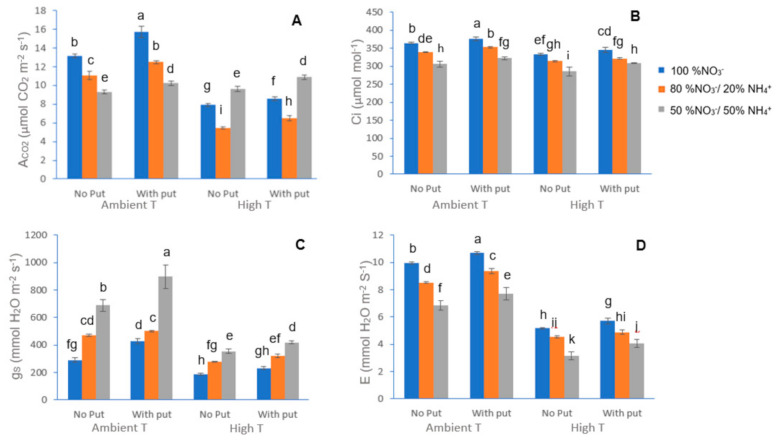
Effect of heat stress, different NO_3_^−^/NH_4_^+^ ratio and absence or putrescine sprayed at 2.5 mM on net photosynthetic rate (A_CO2_) (**A**), intercellular CO_2_ concentration (Ci) (**B**), stomatal conductance (g_s_) (**C**) and evapotranspiration (E) (**D**) in leaves of cauliflower. *n* = 5, ±S.E. Different letters are significantly different according to Tukey test at *p* ≤ 0.05.

Under heat stress conditions, and as the concentration of NH_4_^+^ increased in the nutrient solution, plants showed lower net photosynthetic rate (A_CO2_), intercellular CO_2_ concentration (Ci), and evapotranspiration rate (E) and higher stomatal conductance (g_S_) than control plants (Figure 1A–D). A similar trend was found by others authors when plants were exposed to several abiotic stresses and when the nutritional solution was enriched with NH_4_^+^ [24,25].

It is known that plants in unstressed conditions absorb nitrogen mainly in its ammonium and nitrate forms, both the form of available nitrogen and the amount absorbed by the plant have an important effect on photosynthesis, growth and quality of the plant [15]. Previous works have shown a higher tolerance to abiotic stresses in different plant species fed with different ammonium ratios [26,27]. Wise et al. [28] reported that the reduction of photosynthetic activity in plants was an important factor in the sensitivity of the plant to high temperatures. Thus, it is known that, under high temperatures, plants may close their stomata in order to stay hydrated at the cost of reduced levels of CO_2_ uptake, which affects the rate of photosynthesis [29]. However, the reduction of photosynthetic activity resulting from heat stress is not only attributed to stomatal closure, but also to other factors such as the structural rearrangement of thylakoids [30]. While it is true that the inhibition of photosynthesis with ammonia feeding could be due to ammonium toxicity, it has been reported that ammonium-fed plants could accumulate ammonia in their leaves, which can generate an uncoupling of electron transport in the photophosphorylation process carried out in the chloroplasts, leading to a decrease in the rate of photosynthesis [24]. The lower reduction of stomatal conductance and evapotranspiration found with the 50/50 NO_3_^−^/NH_4_^+^ ratio, as compared to other ratios can be attributed to a higher photosynthesis rate. That increase in photosynthesis could be due to the increase in the activity of antioxidative enzymes during heat stress [31]. As observed in Figure 1A–D, our results showed that a foliar putrescine application resulted in an increase in photosynthesis, as well. This increase in photosynthesis can be explained due to the important role of H_2_O_2_, the product of the oxidation of polyamines by amine oxidases such as copper amine oxidase (CuAO), diamine oxidase (DAO), and polyamine oxidase (PAO), in the sensory transduction process of plants during their responses to biotic and abiotic stress suffered, which affects stomatal closure, which is, in turn, induced by abscisic acid [31].

### 2.2. Chlorophylls and Fluorescence

The results show that the leaf chlorophyll content ranged between 63.93 and 126.55 mg 100 g^−1^ FW (Table 1 and Table 2), whereas no carotene or lycopene were found in cauliflower leaves. As can be observed in Table 1 and Table 2, leaf chlorophyll content in absence of putrescine decreased from 101.11 to 126.55 mg 100 g^−1^ FW in younger leaves and from 63.93 to 98.74 mg 100 g^−1^ FW in outer leaves. Some authors have reported that the reduction of chlorophyll content as consequence of heat stress can be due to either the inhibition of photosynthetic pigment biosynthesis due to oxidative damage generated in thylakoids in plants exposed to high temperature (42 °C), or the chloroplast breakdown as a result of the increased synthesis of reactive oxygen species [32,33,34]. It is known that cauliflower, in order to defend against any kind of stress, use glucosinolates, which are very good antioxidant compounds. Thus, in crucifers, the content of glucosinolates increases in response to heat stress to alleviate its damaging effects [35]. It has been found that these kinds of compounds are higher in crucifer florets than in leaves [36]. It is important to highlight that the glucosinolate content of plants under stress depends on both the level of adaptation to stress and its severity [35]. In this sense, it seems to be that heat stress applied to cauliflower in this work was very severe, because an oxidative process took place. Thus, a consequence of heat stress was a lower content of pigments (chlorophylls). Additionally, Hashimoto et al. [37] reported that a reduction of chlorophyll was linked to protein degradation.

However, the decrease observed due to the action of heat stress was countered by feeding the plants with NO_3_^−^/NH_4_^+^ and spraying them with 2.5 mM putrescine. This was because the application of NO_3_^−^/NH_4_^+^ led to an increase in leaf chlorophyll content, with the highest content achieved with the use of a 50:50 NO_3_^−^/NH_4_^+^ ratio. Additionally, that increase was reinforced with the application of 2.5 mM putrescine (Table 1 and Table 2). Moreover, both the application of NO_3_^−^/NH_4_^+^ and 2.5 mM putrescine also mitigated the decrease observed in the Fv/Fm ratio in cauliflower leaves subjected to heat stress. The observed increase in the photosynthetic parameters in the treatment of 50/50 NO_3_^−^/NH_4_^+^ ratio agrees with the increase obtained in the Fv/Fm parameter, which could be indicative that these plants are favored by a mixed supply of NO_3_^−^/NH_4_^+^ rather than just by nitrate. This could be a possible explanation for why the control value of Fv/Fm is so low [38].

Our results were in agreement with those found in several plants including canola, wheat, and cauliflower, where a remarkable increase in the concentration of photosynthetic pigments was obtained in plants fed with a nutrient solution containing a 50:50 ratio of NO_3_^−^: NH_4_^+^ and with the exogenous application of polyamines, which increased the thermotolerance of a sensitive cultivar [26,31,32]. Particularly, Abd Elbar et al. [32], in a study carried out with *Thymus vulgaris* L., reported that the exogenous application of putrescine alleviated oxidative damage, because it contributed with the maintenance of the thermostability of thylakoid membranes under heat stress.

Regarding the age of the leaves, it is necessary to state that the chlorophyll content gradually decreases as leaf senescence occurs (Table 1 and Table 2). An induced premature senescence is often quantified by a decreasing concentration of chlorophylls and changes in the Chl a/Chl b ratio [39]. Some authors have assured that during senescence not only a reduction of chlorophyll content takes place, but a decrease in the activities of some enzymes such as arginine decarboxylase (ADC) and ornithine decarboxylase (ODC) is also observed, as well as an increase in the activities of polyamine oxidase (PAO) and hydrolases, which accelerates the process of senescence [31]. Additionally, Harding et al. [40] indicated that the senescence of the leaves can be accelerated due to the decomposition of the thylakoid components. Thus, due to the fact that the exogenous application of putrescine can protect the structure of the thylakoid membranes, as shown by the accumulations of not only phenolic compounds but also in the increase in chlorophyll concentrations, some studies have established a relationship between the application of exogenous putrescine and a slowing down of senescence [17,32].

### 2.3. Protein Content

The protein contents of cauliflower cv Moonshine varied significantly, with values between 340.8 and 243.74 g kg^−1^ DW, showing a higher accumulation in the outer leaves. As expected, protein content decreased as temperature increased (Table 3 and Table 4). Similar results were obtained in earlier reports, which showed a reduction of total protein in some plants species under heat stress conditions [34,41,42]. This decrease in protein content could be due to a decrease in the synthesis of proteins or an increase in protease activity, resulting in a higher rate of protein degradation and ultimately to accelerated rate of leaf senescence [13,25,41,43].

As with the chlorophyll content, the total protein content increased in plants fed with a higher NO_3_^−^:NH_4_^+^ ratio, with the maximum protein content observed in the 50/50 NO_3_^−^/NH_4_^+^ ratio treatment (Table 3 and Table 4). Some researchers have found the same results in oilseed rape (*Brassica napus* L.), tomato, and canola [26,44]. In fact, Quin et al. [44] claimed that using nutrient solutions with NO_3_^–^/NH_4_^+^ in an equal ratio promoted the accumulation of soluble proteins. Golvano et al. [45] found that the increase in protein in NH_4_^+^-fed plants was linked to a higher activity of photosynthetic enzymes.

Several studies have reported an enhanced thermotolerance of plants that were exogenously treated with polyamine, through the formation of complexes with antioxidant compounds [31,46]. More specifically, Mostafa et al. [47], in a study carried out with wheat, observed an increase in proteins, one of the signs of mitigating the negative effects of heat stress. This could be mainly attributed to the fact that putrescine can form complexes with proteins, leading to a stable protein structure that prevents their degradation [46]. Additionally, it is interesting to note that polyamines can also promote the inhibition of the movement of the phospholipids in membranes, and stabilize various molecular complexes present in the thylakoid membrane [46]. Additionally, several studies have shown that polyamines, including putrescine, maintain the stability of the membrane during heat stress by reducing electrolyte leakage and lipid peroxidation [17]. However, it is possible that the polyamines do not act separately, but in conjunction with other hormones. In this sense, cytokinins together with polyamines are responsible for regulating physiological and biochemical processes in plants, both under normal conditions and under stress situations. In fact, some authors have indicated that there may be an important correlation between the levels of cytokinins and polyamines when regulating some physiological and biochemical processes in plants, acting as inter and intracellular messengers, which can jointly regulate biotic and abiotic stress. Thus, in this case, it could be that tolerance to heat stress has also been induced by the effect of cytokines [31,48].

As observed in Table 3 and Table 4, different total protein concentrations were found in young and outer leaves. Furthermore, it can be observed that heat stress favored the accumulation of total proteins in young leaves from stressed plants. This is contrasted with the fact that a decrease in total proteins can be used as an indicator of an induced premature senescence [39].

### 2.4. Determination of Lipid Peroxidation

The results of lipid peroxidation showed values between 3.06 and 1.93 TBARS µmol g^−1^ FW (Table 3 and Table 4), being lower in younger leaves from plants sprayed with putrescine and fed with a nutrient solution containing NO_3_^−^/NH_4_^+^ and at ambient temperature. As can be observed in Table 3 and Table 4, lipid peroxidation values increased with increasing temperature, and they decreased in plants fed with nutrient solution containing a NO_3_^−^/NH_4_^+^ ratio of 50:50 and when plants were sprayed with 2.5 mM putrescine. The decrease in lipid peroxidation obtained due to the effect of the 50:50 NO_3_^−^/NH_4_^+^ ratio nutrient solution was consistent with results reported by other authors, who indicated that a nutrition solution with a 50:50 NO_3_^−^/NH_4_^+^ ratio was a good choice for protecting plants against oxidative damage [49]. Although it is true that the exposure of the plants to environmental stresses such as high temperature generates reactive oxygen species, it has been reported that the exposure of the plants to an excess of nitrate or ammonium leads to nutritional stress [50]. Several authors have stated that N-deprived plants generate a greater accumulation of antioxidant compounds, and consequently, a greater protection against peroxidation [15,50]. In another work, the carbon nutrient balance hypothesis has been postulated. According to this hypothesis, in response to nutritional stress, plants accumulate excess carbon, inducing a greater biosynthesis of secondary carbon-based metabolites [15].

The lipid peroxidation decreased after the treatment with putrescine, in agreement with previous findings reported by other authors, who stated that the exogenous application of polyamines mitigates the oxidative damage that appears as a response to different stresses in several plants species (Arabidopsis, wheat, pine, chickpea, *Ganoderma lucidum*, grape, *Thymus vulgaris*) [13,32,41,46]. Lipid peroxidation can be considered as an important index of the damage suffered by the plant under abiotic stress, as it can affect various physiological and biochemical aspects of both plant growth and development [41]. The stress produced in plants after being exposed to high temperatures, induces the generation of reactive oxygen species, which can damage the cell membrane, resulting in lipid peroxidation. For this reason, plants have developed defense mechanisms against this oxidative damage. One of these defense mechanisms consists on the synthesis of protective enzymes (superoxide dismutase (SOD), peroxidase (POD), catalase (CAT), ascorbate peroxidase (APX)) and antioxidant compounds (carotenoids, xanthophylls, polyphenols) in their cell membrane systems [25,50,51]. Thus, a decrease in lipid peroxidation due to the action of the exogenous application of putrescine leads us to consider that putrescine increases the thermotolerance capacity in cauliflower leaves.

Abdallah et al. [52], in a study conducted with lettuce, indicated that abiotic stress induced the lipid peroxidation of only old leaves, whereas younger leaves tended to accumulate antioxidant compounds. This finding is comparable to that observed in cauliflower leaves [53]. Thus, the highest lipid peroxidation was found in the outer leaves. From a nutritional point of view, these by-products antioxidant compounds can be considered important because they contain numerous bioactive compounds with health-promoting properties, such as glucosinolates and phenolic compounds. The literature recognizes them as potential compounds for not only aiding in the prevention, but also as treatment of cardiovascular diseases (hypertension, obesity, arteriosclerosis, and diabetes), and cancers [54,55,56]. Due to the importance of these compounds, in recent decades, different ways have been explored to achieve a greater extraction from plant waste [57,58]. However, thus far, the possibility of achieving a higher concentration of these compounds in agricultural waste products by using different agronomic practices has not been contemplated. In this work, the use of very high temperatures combined with a nutrition solution with a 50:50 NO_3_^−^/NH_4_^+^ ratio and the application of foliar putrescine can lead to a higher and efficient concentration of these secondary metabolites. As a consequence of the concentration of healthy compounds, two beneficial results could be achieved: (i) health-related results; through the use of this N-compound ratio and putrescine, it could be easier to obtain nutraceutical products rich in these compounds, and (ii) sustainability; as the amount of cauliflower residues could be considerably reduced.

### 2.5. Mineral Content

Despite the importance of global warming and with the knowledge that one of its direct effects is thermal stress, there is currently still very little information about how thermal stress produced by high temperatures, chronic or severe, affects the nutrient absorption rate of plants. Our data revealed that cauliflower waste is rich in nutrients (Table 5, Table 6, Table 7 and Table 8). In fact, the concentrations of mineral nutrients observed in cauliflower waste were higher than those observed in florets [53]. This can be ascribed to the fact that crucifer plants under abiotic stress can minimize the concentration of toxic ions in their reproductive organs [36].

As can be observed in our results (Table 5, Table 6, Table 7 and Table 8), the thermal stress caused alterations in the mineral content of the cauliflower leaves. In this sense, a significant decrease was found in practically all the cations (Na, K, Ca, Fe, Zn, Cu, and Mn), and anions (chloride, sulphate and nitrate).

This is consistent with the results found by other authors, who reported that high temperature stress can negatively affect the absorption and accumulation of nutrients in the plant [53,59,60]. More specifically, wheat and maize subjected to high temperatures showed to be Mg-deficient plants [61]. Particularly, a lower content of plant N, a decrease in sulfate and an increase in phosphate were observed in leaves of broccoli and cauliflower under abiotic stress [36,62]. Khalil et al. [61] indicated that heat stress inhibits the movement and transport of nutrients and water both inside and outside the cell. However, the foliar application of putrescine at 2.5 mM resulted, on the one hand, in a significant increase in the concentration of K and nitrates. Similar results were observed in leaves of rose cv. “Dolce Vita”, in melon fruit, and in the cauliflower pellet after the application of exogenous putrescine [53,59,60]. This is because polyamines, among other functions, can block or modulate various types of cation channels. This allows these biogenic amines to control water loss [59,61]. However, on the other hand, it also gave rise to a marked decrease in Na and Cl, and a lower Na/K ratio was also observed as a consequence of the application of putrescine. Our data are consistent with the results found in a pistachio study after the exogenous application of polyamines [63]. Thus, in that study it was shown that polyamines played a very important role in regulating or alleviating the negative effects of abiotic stresses [63].

Regarding the effect of ammonium on the nutrient content of cauliflower leaves, a reduction in the accumulation of some cations (Ca^+^, Mg^2+^, K^+^) and nitrate was observed, as well as an increase in the accumulation of some anions (Cl^−^, SO_4_^2−^ and PO_4_^3−^) (Table 5, Table 6, Table 7 and Table 8). Similar results have been reported by other works carried out with different types of plants [61]. Boxman and Roelofs [64] reported that as a consequence of changes in the one-way influx or efflux of ions, inorganic anion/cation imbalances are produced. Haynes [65] suggested that, in plants that utilize ammonia as their main nitrogen source, changes in the status of different nutrients are observed. These changes do not only occur to overcome inorganic cations/anion imbalances, but are also due to a more complex interaction with other cations, especially potassium.

Different cation and anion levels were observed depending on the age of leaves were studied (Table 5, Table 6, Table 7 and Table 8). Several studies conducted on wheat, cotton, and broccoli showed that old leaves accumulated more anions and cations than new leaves [36,52]. This may be due to the fact that ion accumulate in old leaves in response to the plant’s adaptation to abiotic stress, as several authors reported for sodium and chloride [36,52].

## 3. Material and Methods

### 3.1. Experimental Conditions, Plant Material, and Treatments

Cauliflower (*Brassica oleracea* var. botrytis L.) plants, cv. Moonshine, were obtained from a commercial seed supplier (El Jimenado S.A., Murcia, Spain) 30 days after their germination. Seedlings of similar size were transplanted into 5 L black pots filled with coconut fiber (Pelemix, Alhama de Murcia, Spain) and washed with 2 L of tap water. A total of sixty plants, one plant per pot, were used for carrying out this experiment and they were irrigated with different modified Hoagland solutions. The nutrition solution of the control treatment contained the following: Ca (NO_3_)_2_ 4H_2_O: 362.0 mg L^−1^; KNO_3_: 404.4 mg L^−1^; K_2_SO_4_: 131.1 mg L^−1^; MgSO_4_ 7H_2_O: 123.2 mg L^−1^; H_3_PO_4_: 0.101 mL. The nitrogen contribution in the other two treatments was in the form of NO_3_^−^/NH_4_^+^. Ammonium was introduced as (NH_4_)_2_SO_4_ (105.6 mg L^−1^ and 264 mg L^−1^ for the 80/20 NO_3_^−^/NH_4_^+^ ratio treatment and the 50/50 NO_3_^−^/NH_4_^+^ ratio treatment, respectively). A total of twenty plants per nutrient treatment were used. The nutrients solutions used in the experiment were applied by self-compensating drippers (2 h L^−1^) and drainage was evaluated daily to ensure drainage greater than 35% and for avoiding salt accumulation in the substrate. The experiment was conducted in a climate-controlled chamber designed by our department [66] and located in Murcia, Spain (37°56′27.3″ N, 1°08′01.8″ W). In this chamber, all the environmental conditions were fully controlled: 60% RH, 400 µmol mol^−1^ of CO_2_, 16/8 h day/night, 28/16 °C during the first eighty-six days, and 43/30 °C on the last three days (heat stress). A photosynthetically active radiation (PAR) of 250 µmol m^−2^ s ^−1^ was utilized, provided by a combination of fluorescent lamps (TL-D Master reflex 830 and 840, Koninklijke Philips Electronics N.V., from the Netherlands) and high-pressure lamps (Son-T Agro, Philips).

After eighty-six days, thirty plants (being five plants per treatment) were randomly selected and foliarly sprayed with 20 mL of a solution containing 2.5 mM putrescine plus 0.01% Tween-20 as a surfactant. After 4 days, half of the sprayed cauliflowers and half of the non-sprayed cauliflowers were harvested. The plants that were still in the chamber suffered the heat stress for three days. In total, the experiment lasted ninety-three days after transplantation.

### 3.2. Gas Exchange

Both in the ninetieth day and the final day of the total experiment, the net CO_2_ assimilation rate (A_CO2_), internal CO_2_ concentration (Ci), stomatal CO_2_ conductance (g_s_), and evapotranspiration (E) were measured on the mature fully-expanded leaf of each plant, using a CIRAS-2 portable photosynthesis system (PP system, Amesbury, MA, USA) with a PLC6 (U) Automatic Universal Leaf Cuvette, measuring both sides of the leaves. The cuvette provided light (LED) with a photon flux of 1300 µmol m^−2^ s^−1^, 400 µmol mol^–1^ CO_2_ and a leaf temperature of 25 °C.

### 3.3. Chlorophyll Content and Maximum Potential Quantum Efficiency of PSII

Chlorophylls a and b (Chl a and Chl b) were extracted from 1 g of frozen cauliflower leaves (−80 °C) with 25 mL of extraction buffer (acetone–hexane (2:3)). Leaf samples were homogenized using a Polytron and centrifuged at 3500 rpm for 6 min, at 4 °C. Afterwards, the absorbance of the supernatant was measured at 663, 645, 505, and 453 nm with a spectrophotometer (Shimadzu UV-1800 model with the CPS-240 cell holder, Shimadzu Europa GmbH, Duisburg, Germany). The contents of chlorophylls a and b, lycopene, and β-carotene were determined by using the Nagata and Yamashita [67] equations:

Chlorophyll a (mg 100 mL^−1^) = 0.999 × A663−0.0989 × A645

Chlorophyll b (mg 100 mL^−1^) = −0.328 × A663 + 1.77 × A645

Lycopene (mg 100 mL^−1^) = −0.0458 × A663 + 0.204 × A645 + 0.372 × A505 − 0.0806 × A453

β-Carotene (mg 100 mL^−1^) = 0.216 × A663−1.22 × A645−0.304 × A505 + 0.452 × A453

On the same leaf used for gas exchange, the maximum potential quantum efficiency of PSII (Fv/Fm) was determined following the procedure previously described by Piñero et al. [68]. These measurements were performed by using a portable modulated fluorometer OS-30P (Opti-Science, Hudson, NH, USA). Fv is the variable fluorescence from a dark-adapted leaf and Fm is the maximal fluorescence from a dark-adapted, mature fully expanded leaf. A special leaf clip holder was allocated to each leaf to maintain dark conditions for at least 30 min before reading. The analyses of chlorophyll, lycopene, β-Carotene, and Fv/Fm were run in five repetitions per treatment.

### 3.4. Determination of Total Protein

The total protein was measured in freeze-dried cauliflower leaves that had been dried at 65 °C, for at least 72 h. The analyses (of five replicates per treatment) were performed using a combustion nitrogen/protein analyzer (LECO FP-528, Leco Corporation, St. Joseph, MI, USA).

### 3.5. Lipid Peroxidation

Lipid peroxidation was measured through the thiobarbituric acid (TBA) reaction [69]. Briefly, 0.1 g of lyophilized leaf samples were homogenized with 3 mL of extraction buffer (trichloroacetic acid (TCA), 20% (*w/v*)) and centrifuged at 3500× *g* for 20 min. After, 1 mL of supernatant was mixed with 1 mL of TCA (20%, *w/v*) containing TBA (0.5%, *w/v*) and 150 µL of BHT (4%, *w/v*) in ethanol. This mixture, after being heated for 30 min at 95 °C, was cooled on ice and then centrifuged at 10,000× *g* for 15 min. Thereafter, the absorbance was measured at 532 nm. The value for non-specific absorption at 600 nm was subtracted. The concentration of thiobarbituric acid-reactive substances (TBARS) was calculated using an extinction coefficient of 155 mM^−1^ cm^−1^ [70]. Results were expressed as TBARS µmol g^−1^ FW. The analyses of lipid peroxidation were run in five replicates per treatment.

### 3.6. Leaf Mineral Content

The cauliflower leaf cations (Ca, K, Mg, B, Cu, Fe, Mn, and Zn) were extracted from ground lyophilized material (0.1 g) by acid digestion, using an ETHOS ONE microwave digestion system (Milestone Inc., Shelton, CT, USA), and were analyzed with an inductively-coupled plasma (ICP) spectrometer (Varian Vista MPX, Palo Alto, CA, USA).

The cauliflower leaf anions (Cl^−^, NO_3_^−^, PO_4_^3−^ and SO_4_^2−^) were extracted from ground lyophilized material (0.4 g) with double-distilled water (20 mL) and analyzed with ion chromatography (METROHM 861 Advanced Compact IC; METROHM 838 Advanced Sampler). The analyses of cations and anions were run in five replicates per treatment.

### 3.7. Statistical Analysis

The experimental design was completely random, and five repetitions were carried out. Data were analyzed with SPSS v.21 (IBM, Chicago, IL, USA). First, data were tested for homogeneity of variance and normality of distribution and later, data were also subjected to a three-way (factor 1 = putrescine treatment; factor 2 = heat treatment; factor 3 = nutrition treatment) analysis of variance (ANOVA) and afterwards Tukey’s multiple-range test was utilized to compare the means. Differences were considered statistically significant at *p* ≤ 0.05.

## 4. Conclusions

From the results presented herein, it can be concluded that the cauliflower waste was richer in mineral nutrients than floret cauliflower. This is the first time that the strategy constituted of the application of putrescine and the appropriate ratio of nitrogen forms is proposed in order to improve not only the quality, but also the physiological changes in cauliflower. In this sense, in this work has been seen that putrescine and the appropriate ratio of nitrogen forms showed a synergistic effect on the enhancement of the tolerance capacity of the cauliflower cv Moonshine under heat stress. In fact, the results obtained in this study indicated a higher photosynthesis rate, a higher accumulation of both photosynthesis-related compounds and pigments, total proteins, and a change in the status of the different nutrients due to the application of 2.5 mM putrescine and a nutrition solution with a 50:50 NO_3_^−^/NH_4_^+^ ratio. For this, the application of both putrescine and fertilizing the plants with a 50:50 NO_3_^−^/NH_4_^+^ ratio could be proposed as an agricultural practice that is useful for increasing the thermotolerance of cauliflower cv Moonshine. Regarding the mineral content, it has been seen that under heat stress the content of cations, including Na, K, Ca, P, Fe, Zn, Cu, and Mn, and in anions, including chloride, sulphates and nitrate was decreased. Besides, some cations (Ca^+^, Mg^2+^, K^+^) and nitrate and some anions (Cl^−^, SO_4_^2−^ and PO_4_^3−^) were increased in response to the ammonium effect. It could be as a consequence of changes in the one-way influx or efflux of ions and/or due to a more complex interaction with other cations, especially potassium.

Furthermore, our results suggest that this higher thermotolerance acquired may be correlated with a higher activity of antioxidant enzymes, which reduce lipid peroxidation. Lastly, due to decreased senescence, a lower lipid peroxidation was observed in the young leaves. Additionally, these beneficial effects are not only good for plants, but for humans as well. The occurrence of less lipid peroxidation in cauliflower leaves led to a higher accumulation of secondary metabolites with many health-promoting effects for humans. Therefore, the application of both treatments could be considered as a way to concentrate the content of these metabolites, especially in young cauliflower leaves. Thus, it could be easier to obtain ointments or nutraceutical products starting from young cauliflower leaves rich in these secondary metabolites. A very positive effect of using cauliflower leaves for producing nutraceutical products could be the increased sustainability of cauliflower cultivation.

## Figures and Tables

**Table 1:** Effect of foliar application of putrescine on concentrations of chlorophylls (mg 100 g^−1^ FW) in younger leaves of the cauliflower cv. Moonshine at different ration of NO_3_^−^/NH_4_^+^ and temperatures.

Temperature	NO_3_^−^/NH_4_^+^		Chl a	Chl b	Chl a + Chl b	Chl a/Chl b
Ambient temperature	100/0	Without Put	43.69 ± 1.14 ^ab^	58.37 ± 6.05 ^b^	112.07 ± 7.05 ^de^	0.92 ± 0.19 ^d^
		With Put	53.13 ± 0.55 ^ab^	64.71 ± 8.63 ^a^	117.84 ± 9.00 ^bc^	0.81 ± 0.06 ^e^
	80/20	Without Put	54.03 ± 0.61 ^ab^	60.13 ± 4.38 ^b^	114.16 ± 4.89 ^cd^	0.90 ± 0.14 ^d^
		With Put	54.43 ± 0.23 ^ab^	65.75 ± 8.67 ^a^	120.18 ±8.73 ^ab^	0.83 ± 0.03 ^e^
	50/50	Without Put	54.66 ± 0.65 ^a^	60.91 ± 6.57 ^b^	115.57 ± 7.08 ^cd^	0.90 ± 0.10 ^d^
		With Put	52.03 ± 1.67 ^abc^	74.52 ± 5.48 ^a^	126.55 ± 7.00 ^a^	0.70 ± 0.01 ^f^
High temperature	100/0	Without Put	53.09 ± 1.75 ^ab^	48.02 ± 0.22 ^c^	101.11 ± 3.87 ^hi^	1.11 ± 0.13 ^a^
		With Put	52.82 ± 1.01 ^ab^	53.20 ± 0.22 ^c^	106.03 ± 3.12 ^fg^	1.01 ± 0.21 ^b^
	80/20	Without Put	54.66 ± 1.94 ^a^	47.11 ± 0.99 ^c^	101.78 ± 2.88 ^gh^	1.16 ± 0.18 ^a^
		With Put	51.11 ± 1.67 ^c^	58.20 ± 6.06 ^b^	109.31 ± 7.57 ^ef^	0.87 ± 0.06 ^d^
	50/50	Without Put	53.20 ± 2.41 ^ab^	49.57 ± 2.64 ^c^	102.77 ± 4.95 ^gh^	1.07 ± 0.13 ^a^
		With Put	53.70 ± 1.95 ^ab^	56.62 ± 1.90 ^b^	110.39 ± 3.78 ^ef^	0.94 ± 0.11 ^c^
**Main effects**						
Temperature (T)			***	***	***	***
Putrescine (Put)			***	***	***	***
Nitrate/ammonia (NO_3_^−^/NH_4_^+^)			*	***	ns	*
T X Put			ns	ns	ns	ns
T X NO_3_^−^/NH_4_^+^			ns	ns	ns	ns
Put X NO_3_^−^/NH_4_			ns	ns	ns	ns
T X Put X NO_3_^−^/NH_4_^+^			*	ns	ns	*

Different small letters within a column indicate significant differences among different treatments of putrescine treatments with different NO_3_^−^/NH_4_^+^ ratio and temperatures (*p* = 0.05. Tukey test). *n* = 5, ±S.E. Analysis of variance: ns. not significant; * *p* ≤ 0.05; *** *p* ≤ 0.001.

**Table 2:** Effect of foliar application of putrescine on concentrations of chlorophylls (mg 100 g^−1^ FW) in outer leaves of the cauliflower cv. Moonshine at different ration of NO_3_^−^/NH_4_^+^ and temperatures.

Temperature	NO_3_^−^/NH_4_^+^		Chl a	Chl b	Chl a + Chl b	Chl a/Chl b	F_v_/F_m_
Ambient temperature	100/0	Without Put	54.28 ± 2.27 ^b^	32.37 ± 2.01 ^c^	86.65 ± 4.20 ^c^	1.68 ± 0.09 ^b^	0.776 ± 0.001 ^d^
		With Put	51.11 ± 1.67 ^c^	44.37 ± 1.85 ^a^	95.48 ± 3.45 ^a^	1.15 ± 0.05 ^d^	0.789 ± 0.001 ^b^
	80/20	Without Put	52.82 ± 1.61 ^c^	41.13 ± 1.41 ^b^	93.95 ± 2.96 ^ab^	1.28 ± 0.03 ^c^	0.778 ± 0.001 ^d^
		With Put	53.70 ± 1.14 ^b^	43.34 ± 0.34 ^a^	97.03 ± 1.44 ^a^	1.24 ± 0.06 ^c^	0.793 ± 0.001 ^ab^
	50/50	Without Put	55.29 ± 8.60 ^a^	41.18 ± 1.40 ^b^	96.47 ± 2.22 ^a^	1.34 ± 0.05 ^c^	0.782 ± 0.003 ^c^
		With Put	53.13 ± 0.55 ^b^	45.62 ± 1.85 ^a^	98.74 ± 2.35 ^a^	1.16 ± 0.03 ^d^	0.795 ± 0.001 ^a^
High temperature	100/0	Without Put	42.67 ± 1.91 ^e^	21.27 ± 1.08 ^d^	63.93 ± 2.93 ^d^	2.01 ± 0.03 ^a^	0.746 ± 0.002 ^i^
		With Put	48.93 ± 4.08 ^d^	31.42 ± 1.29 ^c^	80.36 ± 5.56 ^bc^	1.56 ± 0.08 ^c^	0.768 ± 0.001 ^fg^
	80/20	Without Put	43.25± 2.03 ^f^	22.92 ± 2.02 ^d^	66.17 ± 3.96 ^d^	1.89 ± 0.15 ^a^	0.760 ± 0.004 ^h^
		With Put	50.16 ± 3.48 ^d^	31.21 ± 1.05 ^c^	81.36 ± 4.44 ^c^	1.61 ± 0.11 ^bc^	0.769 ± 0.001 ^ef^
	50/50	Without Put	45.28 ± 0.77 ^e^	23.29 ± 0.38 ^d^	68.56 ± 1.13 ^d^	1.94 ± 0.05 ^a^	0.765 ± 0.001 ^g^
		With Put	52.03 ± 1.67 ^d^	31.70 ± 0.55 ^c^	83.73 ± 2.18 ^c^	1.64 ± 0.01 ^b^	0.773 ± 0.001 ^e^
**Main effects**							
Temperature (T)			***	***	***	***	***
Putrescine (Put)			***	***	***	***	***
NO_3_^−^/NH_4_^+^			*	***	ns	*	***
T X Put			***	*	***	*	***
T X NO_3_^−^/NH_4_^+^			ns	**	ns	**	***
Put X NO_3_^−^/NH_4_			ns	***	ns	***	***
T X Put X NO_3_^−^/NH_4_^+^			ns	**	ns	**	***

Different small letters within a column indicate significant differences among different treatments of putrescine treatments with different NO_3_^−^/NH_4_^+^ ratio and temperatures (*p* = 0.05. Tukey test). *n* = 5, ±S.E. Analysis of variance: ns. not significant; * *p* ≤ 0.05; ** *p* ≤ 0.005; *** *p* ≤ 0.001.

**Table 3:** Effect of foliar application of putrescine on lipid peroxidation (TBARS µmol g^−1^ FW) and proteins (g kg^−1^ DW) in younger leaves of the cauliflower cv. Moonshine at different ration of NO_3_^−^/NH_4_^+^ and temperatures.

Temperature	NO_3_^−^/NH_4_^+^		Lipid Perox	Protein
Ambient temperature	100/0	Without Put	2.34 ± 0.01 ^f^	300.50 ± 0.5 ^d^
		With Put	2.07 ± 0.03 ^h^	304.8 ± 0.4 ^bc^
	80/20	Without Put	2.29 ± 0.03 ^f^	301.6 ± 0.1 ^d^
		With Put	2.01 ± 0.02 ^i^	308.3 ± 1.4 ^ab^
	50/50	Without Put	2.21 ± 0.04 ^g^	303.0 ± 0.5 ^c^
		With Put	1.93 ± 0.03 ^j^	311.8 ± 1.1 ^a^
High temperature	100/0	Without Put	2.64 ± 0.01 ^a^	243.74 ± 13.4 ^i^
		With Put	2.53 ± 0.01 ^cd^	284.9 ± 0.2 ^fg^
	80/20	Without Put	2.61 ± 0.01 ^ab^	274.5 ± 3.7 ^h^
		With Put	2.47 ± 0.02 ^de^	287.0 ± 1.5 ^f^
	50/50	Without Put	2.57 ± 0.02 ^bc^	282.3 ± 1.6 ^g^
		With Put	2.40 ± 0.02 ^e^	293.3 ± 1.4 ^e^
**Main effects**				
Temperature (T)			***	***
Putrescine (Put)			***	***
Nitrate/ ammonia (NO_3_^−^/NH_4_^+^)			***	***
T X Put			***	***
T X NO_3_^−^/NH_4_^+^			ns	***
Put X NO_3_^−^/NH_4_			ns	***
T X Put X NO_3_^−^/NH_4_^+^			ns	***

Different small letters within a column indicate significant differences among different treatments of putrescine. treatments with different NO_3_^−^/NH_4_^+^ ratio and temperatures (*p* = 0.05. Tukey test). *n* = 5, ±S.E. Analysis of variance: ns. not significant; *** *p* ≤ 0.001.

**Table 4:** Effect of foliar application of putrescine on lipid peroxidation (TBARS µmol g^−1^ FW) and proteins (g kg^−1^ DW) in outer leaves of the cauliflower cv. Moonshine at different ration of NO_3_^−^/NH_4_^+^ and temperatures.

Temperature	NO_3_^−^/NH_4_^+^		Lipid Perox	Protein
Ambient temperature	100/0	Without Put	2.85 ± 0.01 ^d^	330.0 ± 0.7 ^d^
		With Put	2.72 ± 0.01 ^f^	334.7 ± 0.5 ^b^
	80/20	Without Put	2.79 ± 0.02 ^e^	332.0 ± 0.6 ^c^
		With Put	2.70 ± 0.01 ^f^	339.0 ± 1.0 ^a^
	50/50	Without Put	2.75 ± 0.01 ^e^	333.6 ± 0.3 ^b^
		With Put	2.67 ± 0.01 ^g^	340.8 ± 0.8 ^a^
High temperature	100/0	Without Put	3.06 ± 0.02 ^a^	313.8 ± 0.6 ^h^
		With Put	2.92 ± 0.01 ^c^	323.3 ± 0.5 ^f^
	80/20	Without Put	3.01 ± 0.02 ^b^	316.0 ± 1.2 ^h^
		With Put	2.90 ± 0.01 ^c^	326.7 ± 1.0 ^ef^
	50/50	Without Put	2.94 ± 0.01 ^c^	322.6 ± 0.2 ^g^
		With Put	2.86 ± 0.01 ^d^	328.7 ± 0.2 ^e^
**Main effects**				
Temperature (T)			***	***
Putrescine (Put)			***	***
Nitrate/ ammonium (NO_3_^−^/NH_4_^+^)			***	***
T X Put			ns	***
T X NO_3_^−^/NH_4_^+^			**	***
Put X NO_3_^−^/NH_4_			*	***
T X Put X NO_3_^−^/NH_4_^+^			ns	***

Different small letters within a column indicate significant differences among different treatments of putrescine. treatments with different NO_3_^−^/NH_4_^+^ ratio and temperatures (*p* = 0.05. Tukey test). *n* = 5, ±S.E. Analysis of variance: ns. not significant; * *p* ≤ 0.05; ** *p* ≤ 0.005; *** *p* ≤ 0.001.

**Table 5:** Effect of foliar application of putrescine (2.5 mM) on cations in younger leaves of the cauliflower cv. Moonshine at different ration of NO_3_^−^/NH_4_^+^ and temperatures.

Temperature	NO_3_^−^/NH_4_^+^		Macroelements (g kg^−1^ DW)				Microelements (mg kg^−1^ DW)				
			Na	K	Ca	Mg	Fe	Cu	Mn	Zn	B
Ambient T	100/0	No Put	1.5 ± 0.01 ^a^	13.0 ± 0.1 ^e^	3.5 ± 0.09 ^d^	1.0 ± 0.01 ^e^	15.3 ± 0.5 ^d^	0.7 ± 0.02 ^e^	4.9 ± 0.10 ^e^	43.0 ± 0.6 ^d^	10.8 ± 0.2 ^e^
		With Put	1.2 ± 0.01 ^e^	14.6 ± 0.1 ^a^	4.6 ± 0.02 ^a^	1.2 ± 0.01 ^a^	19.1 ± 0.4 ^a^	1.0 ± 0.02 ^a^	6.5 ± 0.06 ^a^	49.2 ± 0.6 ^a^	12.9 ± 0.4 ^a^
	80/20	No Put	1.4 ± 0.01 ^b^	12.6 ± 0.1 ^f^	3.3 ± 0.03 ^e^	0.9 ± 0.01 ^f^	14.1 ± 0.4 ^e^	0.7 ± 0.01 ^e^	4.8 ± 0.06 ^e^	41.9 ± 0.4 ^e^	10.6 ± 0.1 ^e^
		With Put	1.1 ± 0.01 ^f^	14.4 ± 0.1 ^a^	4.5 ± 0.05 ^a^	1.2± 0.03 ^a^	17.7 ± 0.2 ^b^	0.9 ± 0.01 ^b^	6.4 ± 0.11 ^a^	48.2 ± 0.5 ^ab^	12.5 ± 0.2 ^a^
	50/50	No Put	1.4 ± 0.02 ^b^	12.2 ± 0.1 ^g^	3.0 ± 0.08 ^f^	0.8 ± 0.01 ^g^	13.3 ± 0.2 ^f^	0.6 ± 0.01 ^f^	4.5 ± 0.13 ^f^	40.0 ± 1.0 ^f^	10.5 ± 0.2 ^e^
		With Put	1.1 ± 0.01 ^f^	14.3 ± 0.1 ^b^	4.3 ± 0.03 ^b^	1.1 ± 0.01 ^b^	17.1 ± 0.2 ^b^	0.9 ± 0.01 ^b^	6.2 ± 0.12 ^b^	47.4 ± 0.3 ^b^	12.4 ± 0.2 ^a^
High T	100/0	No Put	1.3 ± 0.01 ^c^	11.6 ± 0.1 ^h^	2.6 ± 0.08 ^g^	0.8 ± 0.01 g	12.3 ± 0.5 ^g^	0.5 ± 0.02 ^g^	4.2 ± 0.06 ^g^	37.8 ± 0.6 ^g^	9.4 ± 0.5 ^f^
		With Put	1.0 ± 0.01 ^g^	14.2 ± 0.1 ^b^	4.2 ± 0.04 ^b^	1.1 ± 0.01 ^c^	16.7 ± 0.1 ^c^	0.8 ± 0.03 ^c^	5.9 ± 0.09 ^c^	47.1 ± 0.2 ^b^	11.7 ± 0.1 ^b^
	80/20	No Put	1.3 ± 0.01 ^c^	11.0 ±0.1 ^i^	2.4 ± 0.02 ^h^	0.8 ± 0.01 ^h^	11.4 ± 0.2 ^h^	0.5 ± 0.02 ^g^	3.9 ± 0.14 ^h^	36.3 ± 0.9 ^g^	9.2 ± 0.6 ^f^
		With Put	0.9± 0.01 ^h^	13.6 ± 0.1 ^c^	3.9 ± 0.04 ^c^	1.0 ± 0.01 ^d^	16.5 ± 0.2 ^d^	0.8 ± 0.01 ^d^	5.5 ± 0.08 ^cd^	45.6 ± 0.1 ^c^	11.5 ± 0.1 ^cd^
	50/50	No Put	1.3 ± 0.03 ^d^	10.4 ±0.2 ^j^	2.3 ± 0.03 ^h^	0.7 ± 0.02 ^h^	9.7 ± 1.7 ^i^	0.3 ± 0.08 ^h^	3.0 ± 0.21 ^i^	32.9 ± 3.2 ^h^	9.2 ± 0.4 ^f^
		With Put	0.9 ± 0.01 ^h^	13.4 ± 0.1 ^d^	3.8 ± 0.05 ^c^	1.0 ± 0.01 ^d^	16.1 ± 0.3 ^d^	0.8 ± 0.01 ^d^	5.2 ± 0.09 ^d^	43.6 ± 0.2 ^d^	11.5 ± 0.2 ^d^
**Main effects**			***	***	***	***	***	***	***	***	***
Temperature (T)			***	***	***	***	***	***	***	***	***
Putrescine (Put)			***	***	***	***	***	***	***	***	***
NO_3_^−^/NH_4_^+^			***	***	***	***	***	***	***	***	***
T X Put			***	***	***	***	***	***	***	***	ns
T X NO_3_^−^/NH_4_^+^			***	***	**	**	ns	ns	***	*	ns
Put X NO_3_^−^/NH_4_			***	***	ns	ns	*	**	***	ns	ns
T X Put X NO_3_^−^/NH_4_^+^			ns	*	**	**	*	**	**	ns	ns

Different small letters within a column indicate significant differences among different treatments of putrescine. treatments with different NO_3_^−^/NH_4_^+^ ratio and temperatures (*p* = 0.05. Tukey test). *n* = 5, ±S.E. Analysis of variance: ns. not significant; * *p* ≤ 0.05; ** *p* ≤ 0.005; *** *p* ≤ 0.001.

**Table 6:** Effect of foliar application of putrescine (2.5 mM) on cations in outer leaves of the cauliflower cv. Moonshine at different ration of NO_3_^−^/NH_4_^+^ and temperatures.

Temperature	NO_3_^−^/NH_4_^+^		Macroelements (g kg^−1^ DW)				Microelements (mg kg^−1^ DW)				
			Na	K	Ca	Mg	Fe	Cu	Mn	Zn	B
Ambient T	100/0	NoPut	2.4 ± 0.01 ^a^	15.6 ± 0.1 ^d^	5.8 ± 0.04 ^f^	1.5 ± 0.01 ^c^	22.9 ± 0.1 ^e^	1.3 ± 0.02 ^b^	7.6 ± 0.22 ^e^	54.7 ± 1.4 ^d^	15.4 ± 0.7 ^c^
		With Put	2.0 ± 0.01 ^e^	17.0 ± 0.1 ^a^	7.2 ± 0.07 ^a^	1.7 ± 0.01 ^a^	26.8 ± 0.2 ^a^	1.4 ± 0.01 ^a^	9.6 ± 0.16 ^a^	61.3 ± 0.7 ^a^	18.0 ± 0.6 ^a^
	80/20	NoPut	2.4 ± 0.01 ^a^	15.6 ± 0.1 ^d^	5.6 ± 0.07 ^g^	1.5 ± 0.01 ^c^	22.2 ± 0.2 ^f^	1.2 ± 0.01 ^c^	7.5 ± 0.08 ^e^	54.8 ± 0.4 ^d^	15.4 ± 0.4 ^c^
		With Put	2.0 ± 0.01 ^e^	16.8 ± 0.1 ^a^	7.0 ± 0.07 ^b^	1.7 ± 0.01 ^a^	25.7 ± 0.2 ^ab^	1.4 ± 0.01 ^a^	9.3 ± 0.08 ^b^	60.9 ± 0.5 ^a^	17.9 ±0.2 ^a^
	50/50	NoPut	2.3 ± 0.01 ^b^	15.3 ± 0.1 ^e^	5.3 ± 0.02 ^h^	1.5 ± 0.01 ^c^	21.7 ± 0.3 ^f^	1.2 ± 0.02 ^c^	7.4 ± 0.16 ^e^	54.0 ± 0.1 ^d^	15.1 ± 0.1 ^c^
		With Put	1.9 ± 0.01 ^f^	16.3 ± 0.1 ^b^	6.7 ± 0.10 ^c^	1.7 ± 0.01 ^a^	25.2 ± 0.1 ^b^	1.4 ± 0.01 ^a^	9.2 ± 0.21 ^b^	59.9 ± 0.3 ^b^	17.5 ± 0.2 ^a^
High T	100/0	NoPut	2.3 ± 0.04 ^b^	15.2 ± 0.1 ^f^	5.1 ± 0.06 ^i^	1.4 ± 0.02 ^d^	21.3 ± 0.2 ^f^	1.2 ± 0.01 ^d^	6.9 ± 0.13 ^f^	51.6 ± 1.3 ^e^	14.1± 0.4 ^e^
		With Put	1.8± 0.03 ^g^	16.1 ± 0.1 ^b^	6.4 ± 0.05 ^d^	1.6 ± 0.01 ^b^	24.9 ± 0.2 ^b^	1.4 ± 0.01 ^a^	8.4 ± 0.25 ^c^	57.7 ± 1.1 ^c^	16.8 ± 0.1 ^b^
	80/20	NoPut	2.2 ± 0.01 ^c^	15.1 ± 0.1 ^g^	5.0 ± 0.03 ^i^	1.3 ± 0.02 ^e^	20.7 ± 0.2 ^g^	1.1 ± 0.02 ^e^	6.8 ± 0.06 ^f^	51.2 ± 0.6 ^e^	13.7 ± 0.1 ^d^
		With Put	1.7 ± 0.01 ^h^	16.0 ± 0.1 ^c^	6.3 ± 0.01 ^e^	1.6 ± 0.01 ^b^	24.1 ± 0.2 ^c^	1.3 ± 0.02 ^b^	8.1 ± 0.09 ^cd^	57.3 ± 0.1 ^c^	16.6 ± 0.7 ^b^
	50/50	NoPut	2.1 ± 0.02 ^d^	14.9 ± 0.1 ^h^	4.8 ± 0.09 ^j^	1.3 ± 0.01 ^e^	20.3 ± 0.2 ^h^	1.1 ± 0.01 ^e^	6.5 ± 0.15 ^g^	51.0 ± 0.5 ^e^	13.6 ± 0.4 ^d^
		With Put	1.6 ± 0.01 ^i^	15.9 ± 0.1 ^cd^	6.0 ± 0.04 ^e^	1.6 ± 0.01 ^b^	23.4 ± 0.1 ^d^	1.3 ± 0.02 ^b^	8.0 ± 0.20 ^d^	57.1 ± 0.6 ^c^	16.6 ± 0.2 ^b^
**Main effects**											
Temperature (T)			***	***	***	***	***	***	***	***	***
Putrescine (Put)			***	***	***	***	***	***	***	***	***
NO_3_^−^/NH_4_^+^			***	***	***	***	***	***	***	***	***
T X Put			***	***	**	***	ns	***	***	ns	ns
T X NO_3_^−^/NH_4_^+^			***	***	ns	***	ns	ns	ns	ns	ns
Put X NO_3_^−^/NH_4_			***	***	ns	***	**	ns	ns	ns	ns
T X Put X R			ns	ns	***	***	ns	*	ns	ns	ns

Different small letters within a column indicate significant differences among different treatments of putrescine. treatments with different NO_3_^−^/NH_4_^+^ ratio and temperatures (*p* = 0.05. Tukey test). *n* = 5, ±S.E. Analysis of variance: ns. not significant; * *p* ≤ 0.05; ** *p* ≤ 0.005; *** *p* ≤ 0.001.

**Table 7:** Effect of foliar application of putrescine on anions (g kg^−1^ FW) in younger leaves of the cauliflower cv. Moonshine at different ration of NO_3_^−^/NH_4_^+^ and temperatures.

Temperature	NO_3_^−^/NH_4_^+^		Chloride	Nitrate	Phosphate	Sulphate
Ambient T	100/0	No Put	1.03 ± 0.1	7.36 ± 0.3	7.78 ± 0.2 ^h^	6.87 ± 0.1 ^g^
		With Put	1.43 ± 0.1 ^a^	13.81 ± 0.5	11.07 ± 0.1 ^b^	10.05 ± 0.1 ^bc^
	80/20	No Put	1.06 ± 0.1	6.44 ± 0.2	8.28 ± 0.2 ^g^	7.21 ± 0.1 ^g^
		With Put	1.47 ± 0.1 ^a^	12.23 ± 0.5	11.20 ± 0.1 ^ab^	10.22 ± 0.1 ^ab^
	50/50	No Put	1.12 ± 0.1	5.73 ± 0.1	9.75 ± 0.4 ^f^	7.76 ± 0.3 ^f^
		With Put	1.49 ± 0.1 ^a^	11.31 ± 0.1	11.49 ± 0.1 ^a^	10.58 ± 0.1 ^a^
High T	100/0	No Put	0.41 ± 0.1 ^e^	5.25 ± 0.1	3.01 ± 0.3 ^k^	4.25 ± 0.1 ^j^
		With Put	1.17 ± 0.1 ^c^	10.80 ± 0.2	10.14 ± 0.1 ^e^	8.41 ± 0.1 ^e^
	80/20	No Put	0.85 ± 0.1 ^d^	0.34 ± 0.1	4.70 ± 0.1 ^j^	5.43 ± 0.4 ^i^
		With Put	1.30 ± 0.1 ^b^	9.60 ± 0.3	10.51 ± 0.1 ^de^	9.26 ± 0.2 ^d^
	50/50	No Put	0.91 ± 0.1 ^d^	0.11 ± 0.1	5.57 ± 0.4 ^i^	6.25 ± 0.1 ^h^
		With Put	1.37 ± 0.1 ^b^	8.56 ± 0.3	10.64 ± 0.1 ^c^	9.76 ± 0.1 ^c^
**Main effects**			***	***	***	***
Temperature (T)			***	***	***	***
Putrescine (Put)			***	***	***	***
NO_3_^−^/NH_4_^+^			***	***	***	***
T X Put			***	***	***	***
T X NO_3_^−^/NH_4_^+^			***	***	***	***
Put X NO_3_^−^/NH_4_			***	***	***	**
T X Put X NO_3_^−^/NH_4_^+^			***	***	**	ns

Different small letters within a column indicate significant differences among different treatments of putrescine. treatments with different NO_3_^−^/NH_4_^+^ ratio and temperatures (*p* = 0.05. Tukey test). *n* = 5, ±S.E. Analysis of variance: ns. not significant; ** *p* ≤ 0.005; *** *p* ≤ 0.001.

**Table 8:** Effect of foliar application of putrescine on anions (g kg^−1^ FW) in older leaves of the cauliflower cv. Moonshine at different ration of NO_3_^−^/NH_4_^+^ and temperatures.

Temperature	NO_3_^−^/NH_4_^+^		Chloride	Nitrate	Phosphate	Sulphate
Ambient T	100/0	No Put	1.95 ± 0.1 ^e^	18.19 ± 0.1 ^e^	12.09 ± 0.1 ^cd^	13.94 ± 0.1 ^g^
		With Put	2.71 ± 0.1 ^bc^	23.16 ± 0.1 ^a^	12.83 ± 0.1 ^ab^	17.51 ± 0.1 ^b^
	80/20	No Put	2.05 ± 0.1 ^e^	17.96 ± 0.1 ^e^	12.29 ± 0.1 ^cd^	14.44 ± 0.2 ^f^
		With Put	2.82 ± 0.1 ^b^	22.33 ± 0.1 ^b^	12.87 ± 0.1 ^ab^	17.93 ± 0.1 ^b^
	50/50	No Put	2.12 ± 0.1 ^de^	16.88 ± 0.1 ^f^	12.43 ± 0.1 ^bcd^	15.37 ± 0.1 ^e^
		With Put	2.98 ± 0.1 ^a^	21.82 ± 0.1 ^b^	13.09 ± 0.1 ^a^	18.32 ± 0.2 ^a^
High T	100/0	No Put	1.58 ± 0.1 ^f^	16.60 ± 0.4 ^f^	11.74 ± 0.1 ^f^	11.43 ± 0.2 ^j^
		With Put	2.15 ± 0.1 ^d^	20.50 ± 0.2 ^c^	12.51 ± 0.1 ^bc^	16.29 ± 0.1 ^d^
	80/20	No Put	1.63 ± 0.1 ^f^	15.04 ±0.1 ^g^	11.82 ± 0.1 ^e^	12.65 ± 0.1 ^i^
		With Put	2.48 ± 0.1 ^c^	19.88 ±0.3 ^c^	12.61 ± 0.1 ^bc^	16.85 ± 0.1 ^c^
	50/50	No Put	1.70 ± 0.1 ^f^	14.74 ±0.1 ^h^	12.02 ± 0.1 ^de^	13.31 ± 0.1 ^h^
		With Put	2.66 ± 0.1 ^c^	19.21 ± 0.2 ^d^	12.71 ± 0.1 ^bc^	17.19 ± 0.1 ^c^
**Main effects**						
Temperature (T)			***	***	***	***
Putrescine (Put)			***	***	***	***
NO_3_^−^/NH_4_^+^			***	***	***	***
T X Put			ns	**	**	***
T X NO_3_^−^/NH_4_^+^			***	***	ns	***
Put X NO_3_^−^/NH_4_			***	ns	*	***
T X Put X NO_3_^−^/NH_4_^+^			***	***	**	**

Different small letters within a column indicate significant differences among different treatments of putrescine. treatments with different NO_3_^−^/NH_4_^+^ ratio and temperatures (*p* = 0.05. Tukey test). *n* = 5, ±S.E. Values are means ± SE of five plants. Analysis of variance: ns. not significant; * *p* ≤ 0.05; ** *p* ≤ 0.005; *** *p* ≤ 0.001.

## Data Availability

Data is contained within the article.
